# Dairy- and Milk-Inspection1Paper read before the Pennsylvania State Veterinary Medical Society, September 21, 1897.

**Published:** 1898-01

**Authors:** C. Courtney McLean

**Affiliations:** Meadville, Pa.


### DAIRY- AND MILK-INSPECTION.[1]

By C. Courtney McLean, V.S.,
MEADVILLE, PA.

(Concluded from page 752, December, 1897.)

I will give you a synopsis of the methods I succeeded in getting our city board of health to adopt four years ago, and the results have been not only decidedly beneficial to all consumers, but very satisfactory to the vendors, all of whom have taken more or less interest and have shown a decided inclination to profit by the requirements in obtaining knowledge that cannot help but tend to improve everything pertaining to the business of milk-production and selling; and, as a result of our milk-inspection, there is one thing specially worthy of mention, and that is, that we have reduced the mortality from that dreaded disease, cholera infantum, so that during the past two years there has been but one death each year from this disease in our city.

### Board of Health Rules.

### *Duties of the Milk Inspector.*

Rule 9. The Inspector of Milk shall annually inspect all dairies, cows, and appointments for supplying milk for sale in the city, shall test all milk offered for sale and procure an analysis of the same whenever the board may direct, and shall receive for his services such compensation as the Board of Health may direct.

### *Of Milk, Cows, and Dairies.*

Rule 56. All persons intending to sell milk in the city of Meadville must make application to the Inspector of Milk for the inspection of his herd, stable, food and water supplies for herd, and apparatus for gathering and distributing the milk, and upon a favorable certificate from such Inspector, and depositing same with the Secretary of the Board of Health, shall be entitled to a certificate from the Board, that the person, having complied with the rules of inspection, is entitled to the privilege of vending milk in the city of Meadville during the ensuing year.

Rule 57. No person shall sell milk within the city of Meadville until he has exhibited to the Inspector of Milk, appointed by the Board of Health, his cows and stables and the cows and stables of all persons from whom he obtains milk, and received from the said Board and posted up conspicuously in his stand, wagon, or sleigh, or in case of personal delivery, otherwise to exhibit on demand, a certificate showing that the cows are healthy, and the stables, food, and appliances are in good sanitary condition. Any addition of non-inspected cows to the herd of any dealer shall be reported to the Inspector of Milk within ten days, and should any person sell the milk of any cow which has not been inspected and approved, he or she so offending

[1] Paper read before the Pennsylvania State Veterinary Medical Society, September 21, 1897.

shall pay a fine of not less than two nor more than ten dollars for each cow whose milk may have been thus sold.

Rule 58. Every vendor of milk within the city shall have his name plainly and conspicuously placed upon his conveyance or place of business.

Rule 59. Every person making an application for inspection as vender of milk shall make known to the Inspector the number of cows owned by him, if any, the name of the owner and number of cows of all persons from whom he gets milk, and such other information as the Board of Health require. He shall not receive, sell, or offer for sale the milk of any cow condemned at any inspection until such condemnation is removed on subsequent inspection, and this shown by a certificate in writing, made and signed by the Inspector and deposited with the Board of Health.

Rule 60. No dealer in milk, and no servant or agent of such dealer, shall sell, exchange, or deliver, or have in his custody or possession with intent to sell, exchange, or deliver, milk from which the cream or any part thereof has been removed, unless in a conspicuous place above the centre upon the outside of every vessel, can, or package from or in which such milk is sold, the words "Skimmed Milk" are distinctly painted in letters not less than one inch in length.

Rule 61. No person shall sell, or have for sale, in the city of Meadville, any unwholesome, diluted, or adulterated milk, or milk known as "swill milk," or milk containing more than eighty-seven and fifty one-hundredths of watery fluid, or less than twelve and fifty one-hundredths per centum of milk solids, or less than three and one-half per centum butter-fats, or the specific gravity of which, at 60° Fahrenheit, shall not be between one and twenty-nine one-thousandths and one and thirty-three one-thousandths, or milk from cows that, for the most part, are kept tied up in stalls, or that are fed swill, still slops, or other like food.

Rule 62. It shall be the duty of the Inspector of Milk, from time to time, to test all milk offered for sale, and to have the same analyzed whenever the Board shall so direct.

Rule 63. Anyone selling the milk of a cow suffering from sickness or injury, on conviction thereof by any magistrate within the city, shall be fined by the said magistrate not less than ten nor more than one hundred dollars.

Rule 64. No milk which has been watered, adulterated, reduced, or changed in any respect from its natural condition by the addition of any foreign substance, shall be brought into, held, kept, sold, or offered for sale at any place in the city.

Rule 65. No milk shall be sold by any person in whose family or residence there may be anyone sick with a contagious disease, especially diphtheria, scarlet fever, or typhoid fever. Milk-tickets must not be used a second time.

*Application for Inspection.*

To , Inspector of Food and Milk for the City of Meadville, Pa.: Desiring to sell milk to the citizens of Meadville, Pa., I make application to you to inspect my and appointments for the supply of milk, under the Rules and Regulations of the Board of Health of said City.

189

Applicant.

Name of owner
Date of inspection

| No. | Breed. | Age. | Temp. | Pulse. | Resp. | Remarks. | | | | |
|---|---|---|---|---|---|---|---|---|---|---|
| | | | | | | | | | | |

Specific gravity.

Average Butter Fats, M. M.

Average Butter Fats, E. M.

Stable—Cubic Area per Head.

[STUB.]

INSPECTOR'S CERTIFICATE.

*To the Board of Health of the City of Meadville, Pennsylvania:*

This is to certify that on the day of I carefully inspected the herd of cows, stalls, food, and water-supply for herd of and respectfully report as follows:

Name of milk-dealer applying for certificate
Location of dairy
Number of cows inspected
Condition of cows as to cleanliness
Disease
Condition of stalls and sheds
Articles of food used for cows
Water-supply
Condition of apparatus for gathering and distributing milk
Names and residences of persons from whom applicant obtains milk
Number of cows
Health of herd and general sanitary condition of cows, stables, and appointments from which applicant obtains milk

I certify that I made the above report after careful examination of the whole, and recommend that be granted a permit to vend milk in the city, under the rules and regulations of the board.

Inspector.

INSPECTOR'S CERTIFICATE.

*To the Board of Health of the City of Meadville, Pennsylvania:*

This is to certify that on the day of I carefully inspected the herd of cows, stalls, food and water-supply for herd of and respectfully report as follows:

Name of milk-dealer applying for certificate
Location of dairy
Number of cows inspected
Condition of all cows as to cleanliness
Disease
Condition of stalls and sheds
Articles of food used for cows

Water-supply
Condition of apparatus for gathering and distributing milk
Names and residences of persons from whom applicant obtains milk
Number of cows
Health of herd and general sanitary condition of cows, stables, and appointments from which applicant obtains milk

I certify that I made the above report after careful examination of the whole and recommend that be granted a permit to vend milk in the city under the rules and regulations of the Board.

Inspector.

PERMIT.

Be it known that now engaged in or about to engage in the business of vending milk within the limits of the city of Meadville, Pa., having made an application to the Inspector of Food of the Board of Health of said city, for inspection and certificate provided for in the amended Supplementary Rules and Regulations of the Board of Health of the City of Meadville, Pa., and said inspection having been made by said officer, and duly certified to the Board that said has fully and satisfactorily met the requirements of the Board of Health is hereby authorized to sell Milk to the citizens of Meadville, Pa., for one year from the day of unless this permit is sooner revoked by said Board for causes not now apparent.

Issued by order of the Board of Health, this day of A. D. 189

Attest:

Secretary. President.

Listed in Class , Fees, $ Date,
Advanced to Class , Fees

Secretary.

Our inspections consist of an examination of the entire premises, cleanliness of the stables, stalls, and cows; the kind and quality of the food and water; how the milk is cooled, and where stored. We require a special milk-house where nothing else can be kept. We do not allow the old pint and quart ticket to be used any more. The cows must be cleaned daily. The wagons and cans must be clean and in good order. The inspections of milk consist of collecting samples of milk—usually eight ounces, as it requires nearly that amount to float a lactometer of the kind I use after getting the specific gravity. I test for butter-fats, using the Babcock tests, which consist of putting 17.5 c.c. of well-shaken milk in a bottle having a graduated scale on a long neck; then add 17.5 c.c. sulphuric acid and shake till the mixture turns a dark brown or black. Then the bottle or bottles are placed in the machine having receptacles that allow the bottoms to turn out and up, caused by the centrifugal motion from turning a handle. Turn the handle five minutes, during which, from not only the chemical action of the acid but the mechanical action caused by turning, the butter-fats are separated and come to the top of the mixture. Then enough hot water is added to cause the oil to rise into the graduated stem, and you then read the amount of butter-fats the sample contains by the graduated scale.

I evaporate to dryness to ascertain the amount of solids, and I use Hehner and Richmond's table for calculation of total solids from specific gravity and butter-fats.

The detection of adulterations from water, if not told by the lactometer, then use the diphenylamin test.

There are numerous preservalines in the market to keep milk from souring, containing either sodium carbonate, boric acid, or salicylic acid, all of them easy of detection: sodium carbonate, by the rosalic-acid test, boric acid by the flame test, and salicylic acid by solution ferri perchlor. Formaldehyde will, no doubt, be extensively used when its great preservative powers are better known among dealers. The use of any of these preservalines is not so injurious to the milk, but if dealers are allowed to use them, as a matter of course, they will become more negligent and filthy, as they know their milk will keep a long time. There is no necessity for any preservative in milk when properly produced and handled as it should be.

### Five Samples of Franklin Milk Tested.

September 11, 1897.

| | Bottle No. | Butter-fats. | Sp. gravity. | Solids. | Cream by per ct. |
|---|---|---|---|---|---|
| No. 1, | 6 | 4.6 per ct. | 1029 | 12.72 per ct. | 11 per ct. |
| No. 2, | 16 | 3.6 " | 1030 | 11.82 " | 9 " |
| No. 3, | 8 | 3.4 " | 1030 | 11.58 " | 8 " |
| No. 4, | 3 | 3.8 " | 1029 | 12.05 " | 10 " |
| No. 5, | 4 | 4.0 " | 1029 | 12.02 " | 10 " |

### Copy of Standing of Milk Tested.

June 25, 1897.

| Name of dealer. | Sp. gravity. | Butter-fats. | Solids. |
|---|---|---|---|
| Limber, W. . . . | 1028 | 4.0 per ct. | 11.77 per ct. |
| Gleason, F. E. . . . | 1031 | 4.0 " | 12.54 " |
| Ellis, H. E. . . . | 1031 | 4.2 " | 12.77 " |
| Cullum, C. S. . . . | 1028 | 4.3 " | 12.89 " |
| Cotton, H. C. . . . | 1031 | 5.2 " | 13.82 " |
| Weller, J. . . . . | 1031 | 4.4 " | 13.00 " |
| Roha, August . . . | 1031 | 4.4 " | 13.00 " |
| Stewart, J. . . . . | 1031 | 4.2 " | 12.77 " |

| Name of dealer. | Sp. gravity. | Butter-fats. | Solids. |
|---|---|---|---|
| Cotton, Ed. | 1031 | 3.8 per ct. | 12.31 per ct. |
| Deater, S. E. | 1028 | 4.8 " | 12.70 " |
| Sherick, Dan. | 1029 | 4.0 " | 12 02 " |
| Van Horn, B. | 1029 | 4.4 " | 12.49 " |
| Roha, Joseph | 1029 | 4.0 " | 12.02 " |
| Cotton, Fred. | 1029 | 4.2 " | 12.77 " |
| Wagner, H. | 1031 | 4.0 " | 11.56 " |
| Schwabus, A. | 1029 | 4.4 " | 12.49 " |
| Acuff, G. B. | 1029 | 5.0 " | 12.42 " |
| Lenheim, L. | 1026 | 4.6 " | 13.24 " |
| Kightlinger | 1031 | 4.0 " | 12.02 " |
| Hamilton, J. W. | 1031 | 5.0 " | 13.70 " |
| Laver, George | 1031 | 4.2 " | 12.77 " |
| Minium, S. | 1031 | 4.0 " | 12.54 " |
| Roha, Jacob | 1031 | 4.4 " | 12.54 " |
| Onspaugh, W. E. | 1031 | 4.0 " | 12.54 " |

JULY 7, 1897.

| Name of dealer. | Sp. gravity. | Butter-fats. | Solids. |
|---|---|---|---|
| Roha, August | 1030 | 3.8 per ct. | 12.05 per ct. |
| Minium, S. | 1030 | 3.8 " | 12.05 " |
| Laver, George | 1029 | 4.5 " | 12.60 " |
| Stewart, J. | 1030 | 4.2 " | 12.51 " |
| Weller, John | 1029 | 5.0 " | 13.18 " |
| Cotton, Fred. | 1032 | 3.5 " | 12.21 " |
| Cullum, C. S. | 1029 | 4.5 " | 12.60 " |
| Cotton, Ed. | 1030 | 3.8 " | 12.05 " |
| Sherick, D. | 1030 | 4.2 " | 12.51 " |
| Van Horn, B. | 1030 | 4.0 " | 12.28 " |
| Hamilton, J. W. | 1030 | 4.2 " | 12.51 " |
| Ellis, H. E. | 1032 | 3.6 " | 12.33 " |
| Acuff, G. B. | 1030 | 5.0 " | 13.44 " |
| Limber, J. W. | 1029 | 4.4 " | 12.49 " |
| Cotton, H. C. | 1029 | 4.8 " | 12.95 " |
| Deater, S. C. | 1029 | 4.0 " | 12.02 " |
| Roha, Jacob ("Preservative Found.") | 1030 | 3.6 " | 11.82 " |
| Schwabus, A. | 1030 | 3.8 " | 12.05 " |
| Gleason, F. E. | 1030 | 4.0 " | 12.28 " |
| Onspaugh, W. E. | 1030 | 4.2 " | 12.51 " |
| Kightlinger | 1030 | 4.0 " | 12.28 " |
| Lenheim | 1029 | 4.0 " | 12.02 " |
| Roha, Joseph | 1029 | 4.6 " | 12.72 " |

AUGUST 11, 1897.

| Name of dealer. | Sp. gravity. | Butter-fats. | Solids. |
|---|---|---|---|
| Roha, Joseph . . . . | 1029.5 | 3.9 per ct. | 11.69 per ct. |
| Laver, George . . . | 1030.5 | 4.4 " | 12.87 " |
| Roha, Jacob . . . | 1029.5 | 4.0 " | 12.41 " |
| Stewart, J. . . . . | 1029.5 | 5.0 " | 13.31 " |
| Lenheim, L. . . . | 1030.5 | 5.0 " | 13.51 " |
| Hamilton, J. W. . . | 1029.5 | 5.2 " | 13.81 " |
| Kightlinger . . . | 1030.5 | 4.6 " | 13.11 " |
| Schwabus, A. . . . | 1029.5 | 4.2 " | 12.38 " |
| Cotton, Ed. . . . | 1029.5 | 4.6 " | 12.85 " |
| Cotton, Fred. . . . | 1029.5 | 4.6 " | 12.85 " |
| Ellis, H. E. . . . | 1029.5 | 4.2 " | 12.38 " |
| Sherick, D. . . . | 1027.5 | 4.0 " | 11.64 " |
| Acuff, G. B. . . . | 1029.5 | 4.6 " | 12.85 " |
| Van Horn, B. . . . | 1029.5 | 4.2 " | 12.38 " |
| Gleason, F. E. . . . | 1029 | 4.0 " | 12.41 " |
| Cullum, C. S. . . . | 1029 | 4.6 " | 12.85 " |
| Onspaugh, W. E. . . | 1029.5 | 5.0 " | 13.31 " |
| Roha, August . . . | 1029 | 4.2 " | 12.38 " |
| Weller, John . . . | 1029 | 4.6 " | 12.85 " |
| Limber, J. W. . . . | 1029 | 4.2 " | 12.38 " |
| Deater, S. E. . . . | 1029 | 4.6 " | 12.85 " |
| Cotton, H. C. . . . | 1029 | 4.2 " | 12.38 " |

C. COURTNEY MCLEAN,
Inspector for Meadville, Pa., Board of Health.

